# Research on Two-Stage Data Compression at the Acquisition Node in Remote-Detection Acoustic Logging

**DOI:** 10.3390/s25144512

**Published:** 2025-07-21

**Authors:** Xiaolong Hao, Yangtao Hu, Bingnan Yan, Hang Hui, Yunxia Chen, Bingqi Zhang

**Affiliations:** 1 Downhole Measurement and Control Laboratory of National Engineering Laboratory of Oil and Gas Drilling Technology, Xi’an 710065, China; xlhao@xsyu.edu.cn (X.H.); 15594553688@163.com (Y.H.); 23211030343@stumail.xsyu.edu.cn (H.H.); z1033981768@163.com (B.Z.); 2 School of Electronic Engineering, Xi’an Shiyou University, Xi’an 710065, China; 3 China National Logging Corporation, Xi’an 710077, China; chenyx900518@163.com

**Keywords:** acoustic logging, two-stage data compression, wavelet transform, adaptive differential pulse-code modulation, FPGA

## Abstract

The substantial volume of data acquired through remote-detection acoustic logging poses a remarkable challenge because of the limited real-time upload speed of the cable, which severely impedes its further application. To address this issue, a two-stage data compression method that was implemented at the acquisition node was proposed in this study. This approach includes a field programmable gate array (FPGA)-based hardware system and a two-stage downhole data compression algorithm combining wavelet transform and adaptive differential pulse-code modulation paired with ground decompression software. Finally, the proposed compression method was evaluated using actual logging data. The test results revealed that the overall compression rate of the two-stage compression method was 25.1%. The reconstructed waveforms highly retained the overall shape of the original waveforms, and the severe relative distortion of individual data points did not affect the extraction of the sliding longitudinal, sliding transverse and reflected waveforms. The FPGA compressed 2048 16-bit waveforms in approximately 100 μs with low resource utilization and workload. It considerably outperformed DSP-based pre-transmission compression. Herein, the data compression method at the acquisition node helped in reducing the workload on the master control node and increasing the effective speed of the cable transmission up to 400%, thereby enhancing the remote-detection acoustic logging.

## 1. Introduction

Remote-detection acoustic logging is a key technology in oil and gas exploration. It can effectively identify faults, fractures and other geological structures near the borehole and provide extensive information for reservoir assessment and production guidance [1]. However, as the detection range and precision of this logging escalate, the volume of downhole logging data escalates [2,3]. The current cable transmission rate below 1 Mbps finds it difficult to keep up, making it difficult to transmit all the data quickly to the surface and limiting the logging speed to below 120 m/h. Although a “downhole storage, partial uploading” strategy can enhance the logging speed to 400 m/h, it requires all the data to be read from the storage module of the tool when returning to the surface, which does not support real-time analysis at the wellsite [4,5,6]. Downhole data compression offers a promising solution to real-time transmission problems in remote-detection acoustic logging, thus opening up broad application prospects.

Data compression technology is extensively used in various domains, including communications, image processing, video coding, satellite remote sensing, medical imaging and oil and gas exploration [7,8,9,10]. In seismic exploration, Fajardo et al. used a two-dimensional lifting wavelet transform algorithm to compress seismic data to effectively reduce the storage requirements and transmission time [11]. For logging while drilling (LWD), Schlumberger integrated the DigiScope data transmission platform with the Orion II data compression platform, increasing the LWD data transfer rate to 140 bit/s [12]. Erdal et al. integrated wavelet packet decomposition with adaptive thresholding to compress ultrasonic signals [13]. Zhang et al. effectively used adaptive differential pulse-code modulation (ADPCM) to compress relatively smooth gamma-ray and resistivity signals In acoustic LWD [14], Li et al. compressed non-steady LWD acoustic logging signals using a segmented compression model that combines predictive coding and wavelet transform [15]. In open-hole acoustic logging, Zhang compressed acoustic array waveform data using an enhanced SPIHT algorithm, and the execution time per waveform was approximately 6 ms [16]. However, this work only studied the distortion of sliding waves with large amplitudes in the full waveform, and it did not consider the reflection wave with a weak amplitude. Hao et al. applied the DPCM algorithm to remote-detection acoustic logging data, achieving an compression rate of approximately 50% with an execution time of 5–10 ms per waveform [17].

Despite these advancements, most existing downhole data compression methods for acoustic logging run on the master control digital signal processor (DSP), which also handles data uploading and other functions. The use of serial processing by the DSP substantially increases the data execution time of complex compression algorithms. Therefore, downhole tools are limited to simple, single compression algorithms, resulting in low compression rates. Furthermore, compression algorithm execution is time consuming during normal operations, increasing the workload of the DSP and adversely impacting real-time data transmission and other functions.

**Figure 1:**
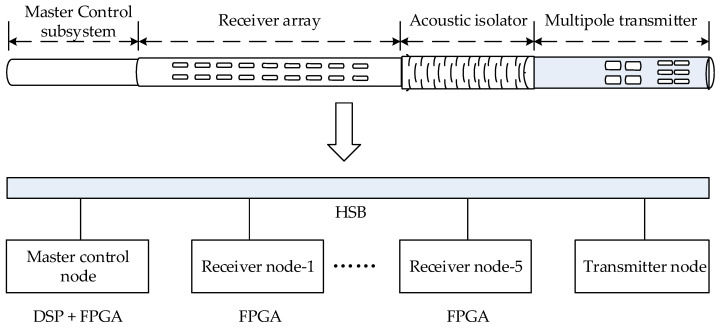
Remote-detection acoustic logging tools and control architecture.

**Figure 2:**
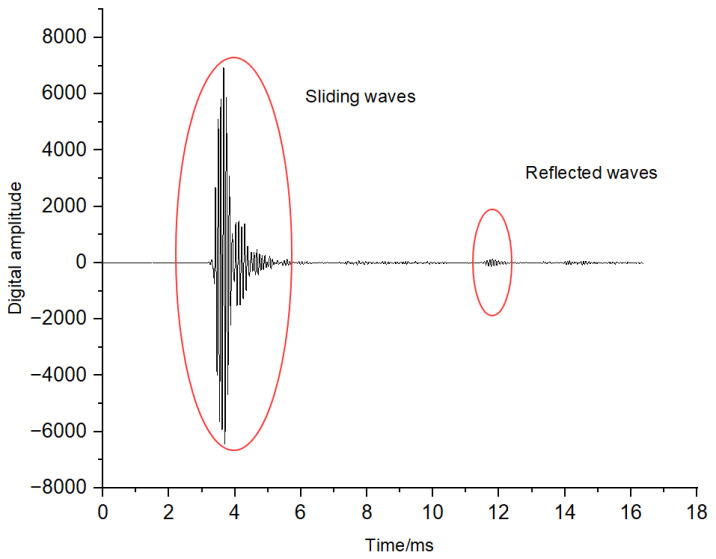
Monopole full waveform acquired during remote-detection acoustic logging.

This issue is especially critical in remote-detection acoustic logging tools, which consist of four subsystems: a master control subsystem, a receiver array, an acoustic isolator, and a multipole transmitter, as shown in the upper part of Figure 1. The transmitter node includes monopole and dipole sources. The receiver array consists of 10 stations with a total of 80 receiving channels. Each station has eight receiving transducers arranged in a circular pattern at intervals of 45°. Every two stations share one receiver node, which uses an FPGA as its acquisition controller. The master control node uses a cooperative DSP and FPGA configuration to manage five receiver nodes and the transmitter node via a high-speed synchronous bus, as shown in the lower part of Figure 1 [18,19]. The tool operates in a “monopole + dipole” mode and uses 16-bit AD converters for sampling. In monopole mode, the transmitter node emits monopole longitudinal waves into the formation, and each receiving channel acquires a full waveform comprising 2048 data points with a sampling interval of 8 μs. In dipole mode, the transmitter node emits dipole waves into the formation, and all 80 channels acquire and synthesize into 40 dipole full waveforms with the same sampling depth and interval. Therefore, during each work cycle, 120 full waveforms are generated, with a total data volume of 480 kB. These full waveforms also display clear nonstationary and abrupt characteristics. Figure 2 shows a typical monopole full waveform, with the main frequency of the useful signal ranging from 1 to 20 kHz. The initial part of the waveform contains the sliding waves used to extract the longitudinal and transverse wave information of the formation, while the latter part contains the reflected waves near the wellbore, which are used to determine the reflector positions [6]. Therefore, the development of downhole data compression methods that can achieve a high compression ratio with low distortion, reduce the workload of the master-controlled DSP and not affect the current functions of the tool is crucial.

To overcome these challenges, this study proposes a two-stage data compression method at the acquisition node for remote-detection acoustic logging. The FPGA-based acquisition controller, the software for two-stage compression and the corresponding ground decompression were developed. The performance of the proposed algorithm was evaluated using actual logging data. This study reduced the workload on the master control node and enhanced the efficiency of the cable transmission, thereby providing a solution for cost reduction and increased efficiency in remote-detection acoustic logging.

## 2. Principle of Two-Stage Compression in Data Acquisition Node

**Figure 3:**
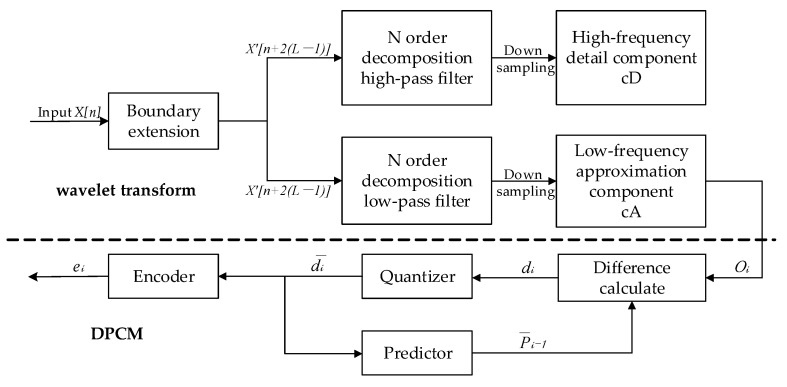
Principle of downhole two-stage compression.

Considering the tool structure and data characteristics, a two-stage compression structure with low distortion and a high compression rate was achieved by combining the wavelet transform with the ADPCM algorithm. Figure 3 illustrates the principle of the two-stage downhole compression. The first stage uses the wavelet transform method to compress the original acoustic logging data, and the second stage uses the ADPCM algorithm to compress the output data from the first stage. Ground decompression is performed in the reverse sequence of the downhole compression.

The first-stage compression module uses a one-layer wavelet transform algorithm, which constructs multi-stage filters with varying cut-off frequencies to decompose non-smooth signals into sub-band components with different frequency bands, highlighting the main energies and eliminating the secondary components [8,20]. The first-stage compression module performs data boundary extension, low-pass filtering and high-pass filtering. To avoid the edge effect from signal truncation on the calculation results, we first perform a boundary symmetric extension on the original logging data *X*[*n*] to *X*[*N* + 2 × (*L* − 1)] (Equation (1)). Here, *N* represents the length of the original signal (2048), and *L* is the length of the wavelet filter (8 for the Daubechies-4 wavelet, DB4). Then, after boundary extension, the data are fed into the low-pass filter *h*[*m*] and high-pass filter *g*[*m*] for band separation. Finally, two-fold downsampling is used on the two sub-band signals from the filter output to obtain the low-frequency approximate component *cA*[*k*] and the high-frequency detail component *cD*[*k*]. Equations (2) and (3) illustrate the calculations for *cA*[*k*] and *cD*[*k*]. The *cA*[*k*] retains the main signal features and is transferred to the second-stage compression module for further processing. Here, the *cD*[*k*], comprising noise and secondary information, is discarded.

(1) $$X^{\prime }(n)=\left\{\begin{array}{ll} X(-n-1) & \;\;-L+1\leq n\leq -1\\ X(n) & \;\;0\leq n\leq N-1\\ X(2N-n-1) & \;\;N\leq n<N+L-1 \end{array}\right.,$$

(2) $$cA[k]=\sum_{m}X^{\prime }(2k-m)\cdot h[m]\;0\leq m<L,k=0,1,2,3,\ldots ,[N+(L-1)]/2,$$

(3) $$cD[k]=\sum_{m}X^{\prime }(2k-m)\cdot g[m]\;0\leq m<L,k=0,1,2,3,\ldots ,[N+(L-1)]/2.$$

The second-stage compression module uses the ADPCM algorithm to reduce the data bits by leveraging data correlation through difference calculation, prediction, quantization and encoding [21,22]. In this design, the low-frequency components from the wavelet transform output serve as the initial input to the original data $O_{i}$. The difference calculation module computes the difference sequence $d_{i}$ (Equation (4)), where ${\bar{P}}_{i-1}$ is the previous prediction value. The quantization module performs non-uniform quantization to convert the difference sequence $d_{i}$ into a distinct quantized value sequence ${\bar{d}}_{i}$, according to the encoding requirements. Equation (5) illustrates the quantization method, where $q_{i}$ is the quantization error. The encoder module uses adaptive encoding to convert the 16-bit original data $O_{0}$ and the quantized value sequence ${\bar{d}}_{i}$ into an 8-bit code sequence $e_{i}.$ The low and high bytes of $O_{0}$ are stored as $e_{o}$ and $e_{n}$, respectively. The remaining bytes of $e_{i}$ are sequentially stored as the encoding result of the quantized value sequence ${\bar{d}}_{i}$. The prediction module generates the current prediction value $P_{i}$ based on the previous prediction value ${\bar{P}}_{i-1}$ and the current quantization value ${\bar{d}}_{i}$ (Equation (6)).

(4) $$d_{i}=O_{i}-{\bar{P}}_{i-1}\;\;\;i=1,2,3,\ldots ,n-1,$$

(5) $${\bar{d}}_{i}=d_{i}+q_{i}\;\;\;\;\;\;\;\;i=1,2,3,\ldots ,n-1,$$

(6) $${\bar{P}}_{i}=\left\{\begin{array}{ll} O_{0} & \;\;i=0\\ {\bar{P}}_{i-1}+{\bar{d}}_{i} & \;\;i=1,2,3,\ldots ,n-1 \end{array}\right..$$

In two-stage data compression, the distortion of the first-stage compression based on the wavelet transform is primarily because of the high-frequency components of the signal. The distortion of the second-stage compression based on the ADPCM is only related to the quantization error $q_{i}$. Equation (7) illustrates the relationship between the reconstructed ${\bar{O}}_{i}$ and original values $O_{i}$.

(7) $${\bar{O}}_{i}=O_{i}+q_{i}.$$

## 3. Realization of Two-Stage Compression in FPGA

As the FPGA is the acquisition controller in the remote-detection acoustic logging receiving node, implementing the two-stage compression algorithm on the FPGA means compression at the acquisition node. The FPGA realization of two-stage compression is illustrated as follows.

### 3.1. Hardware Design

**Figure 4:**
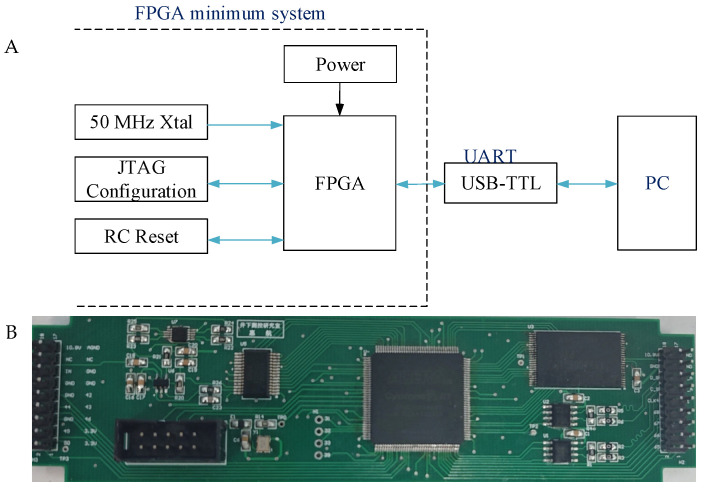
(**A**) Schematic of the hardware system. (**B**) Actual picture of the hardware system.

Figure 4A shows a schematic of the FPGA-based two-stage data compression system. The system comprises an FPGA minimum system and host PC, which are connected through a universal asynchronous receiver–transmitter (UART) serial module. The FPGA minimum system, which is responsible for executing the downhole two-stage compression algorithm, includes the FPGA, power, external crystal, reset and JTAG configuration module [23,24,25]. EP3C25E144I7, which is used in the downhole tool, was chosen as the FPGA. The UART module uses a USB-TTL module to realize bi-directional transmission between the FPGA and the host PC. An actual picture of this hardware system is shown in Figure 4B.

### 3.2. Firmware Design

Herein, the “bottom-layer Verilog HDL + top-layer schematic” method was used to develop the FPGA-based two-stage compression firmware. This development comprises three aspects: first-stage compression based on wavelet transform, second-stage compression based on ADPCM and simulation. Here, we consider the compression of a 2048-word remote-detection acoustic logging waveform as an example to illustrate the realization method.

#### 3.2.1. Realization of the First-Stage Compression

**Figure 5:**
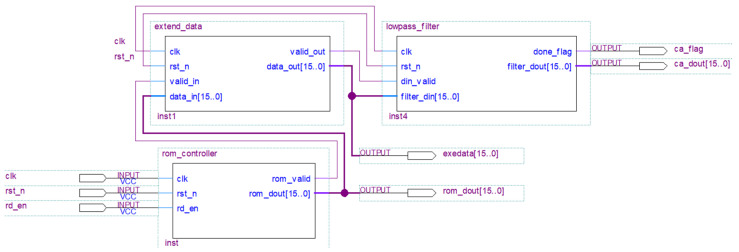
Schematic of the one-layer wavelet transform.

Figure 5 shows the top-level schematic of the one-layer wavelet transform realized in the FPGA, including the data input control module (rom_controller), data extension module (exetend_data) and low-pass filter convolution and downsampling modules (lowpass_filter). The wavelet-transform-based data compression is achieved using the following three modules. The original logging data is saved in the read-only memory (ROM) of the FPGA to verify the effectiveness of the algorithm. However, in actual tools, first-in first-out (FIFO) memory is used to interface the data acquisition and compression modules.

(1) The rom_controller module controls the ROM output, ensuring that the original data are sent to the exetend_data module. The system operates using the 50 MHz system clock (clk) and reset signal (rst_n). When the read enable signal (rd_en) is high, the module reads 16-bit unsigned logging data from the ROM one by one and outputs it via rom_dout[15..0]. The exetend_data module can read the current output data when the valid signal (rom_valid) is high.
(2) The exetend_data module is designed based on the state machine concept, which caches and boundary-extends the data received from the rom_controller module and prepares it for subsequent convolutional processing. When the input valid signal (valid_in) is high, the input data (data_in[15..0]) is cached in the internal RAM and then symmetrically mirrored and extended at both ends using the signal edges as reference points. The symmetric extension transforms the original 2048 data sequence into 2062 data (Equation (1)). Once the extension is completed, the processed data are output via data_out[15..0] after the output valid signal (valid_out) is pulled high.
(3) The lowpass_filter module performs the convolutional computation based on db4 wavelets with a filter length of 8. As the sampling interval is 8 us, the cut-off frequency of the lowpass_filter is 31.25 kHz. This module uses an 8-level data shift register as the front-end caching unit for its convolutional pipeline. When the data valid signal (din_valid) is high, the extended input data (filter_din) are sequentially pushed into the registers in each clock cycle, thereby creating a dynamic sliding window. Once the register groups are filled, the cached data are convolved with integer-based low-pass filter coefficients in a three-stage pipeline. The first stage uses a parallel multiplier for the simultaneous multiplication of the eight data points with their coefficients. The second and third stages sequentially add the eight parallel multiplication results into the final convolutional sum using a two-stage addition pipeline. Combining FIFO storage and a modulo-2 cycle counter that uses conditional enable signals allows for half-extraction data compression reduction and downsampling. After downsampling, the approximate component calculation completion flag (ca_flag) goes high and enables the external modules to sequentially read the low-frequency component sequence cA through the filter_dout interface.

#### 3.2.2. Realization of Second-Stage Compression

**Figure 6:**
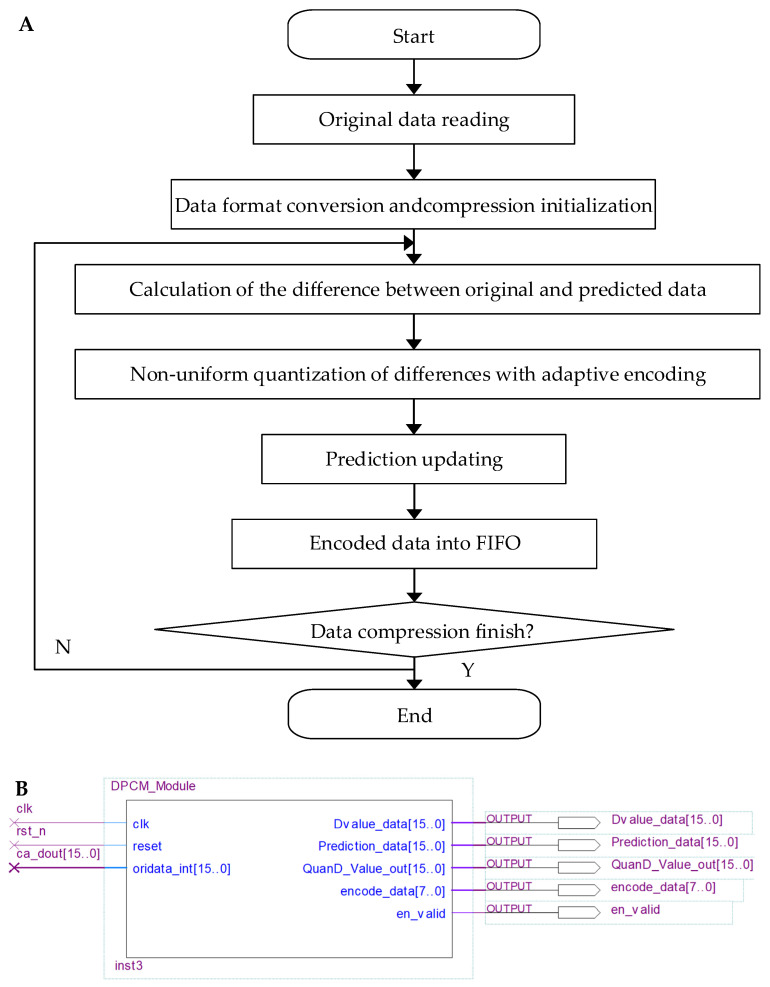
(**A**) Flow diagram and module of the ADPCM compression. (**B**) Schematic of the ADPCM_module.

The second-stage compression uses the ADPCM algorithm. Figure 6A shows a flow diagram of the algorithm’s implementation. The output sequence cA from the first-stage compression serves as the input, producing an 8-bit encoded data sequence (encode_data[7..0]). As the difference calculation, quantization, prediction, encoding and other operations of this algorithm require close cooperation and feedback, we integrate them into the same module (DPCM_Module) (Figure 6B). This allows the algorithm to run efficiently with parallel processing and a pipelined architecture.

During the initialization phase of the reset, the DPCM_Module sets the initial prediction value ${\bar{P}}_{0}$ to the original data $O_{0}$, assigning its lower and higher eight bits to the encoded values $e_{o}$ and $e_{n}$, respectively. Once the reset is complete, the module reads the 16-bit unsigned data output from the first-stage compression one by one through the data input interface (oridata_int[15..0]) with the clock cycle. These data are then converted into 16-bit signed data. Starting from the second clock rising edge, the module calculates the difference between the current data $O_{i}$ and the previous prediction value ${\bar{P}}_{i-1}$. To reduce the distortion, the algorithm performs interval quantization on the absolute value of the difference. The quantized difference is then adaptively encoded into a single 8-bit output data (encode_data[7..0]) based on Table 1. The output format adapts between two encoding modes: it consists of a 1-bit sign code, a 4-bit (or 3-bit) segment code, and a 3-bit (or 4-bit) interval code, depending on the quantization result. This effectively compresses each 16-bit input data into 8-bit output value. Finally, the current prediction value ${\bar{P}}_{i}$ is updated, and the encoded data are stored. To facilitate access by other modules, this design retains the compression results in an FIFO.

#### 3.2.3. Simulation and Analysis

**Figure 7:**
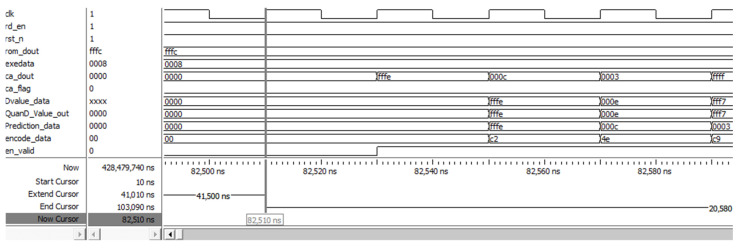
Results of the two-stage compression simulation.

To verify the effectiveness of the two-stage compression, this study simulates the FPGA software (FPGA software ModelSim-Altera 10.4b) using the Modelsim tool. Figure 7 shows the simulation results of the two-stage compression algorithm. The timing waveforms at a specific moment can be observed by pulling the progress bar. Traversing the entire simulation time shows that at time 10 ns (Start Cursor), the system starts to read the original data (rom_dout) from the ROM at a 50 MHz clock signal (clk), guided by the read enable signal (rd_en) and the reset signal (rst_n). At 41,010 ns (at Extend Cursor), once the data reading is completed, the system starts calculating and generating extended data (exedata). At 82,510 ns (at Now Cursor), all the extended data are outputted and the system starts filtering and downsampling under the clock, sequentially outputting the wavelet low-frequency component (ca_dout) data. After one clock of ca_dout starts outputting, the DPCM module starts calculating and generating the difference value (Dvalue_data), quantization value (QuanD_Value_out), prediction value (Predction_data) and encoding value (encode_data) in sequence (Figure 7). Once the wavelet decomposition is complete, the ca_flag for the low frequency of the wavelet components goes high. The data output valid flag (en_valid) turns low when the system runs to 103,090 ns (End Cursor), indicating the completion of the two-stage compression algorithm. The simulation results are as expected and thus confirm the accuracy of the two-stage compression firmware.

## 4. Testing and Analysis

According to the performance tests of the two-level compression algorithm, we analyzed the feasibility of using the algorithm in remote-detection acoustic logging.

### 4.1. Performance Testing

This study tested multi-channel remote-detection acoustic logging data using a full waveform comprising 2048 16-bit data points as an illustrative example. During the testing, the data were compressed in the FPGA in two stages to measure the execution time and resource usage. The data were then sent to a host PC for decompression and evaluation of the distortion and other parameters.

**Figure 8:**
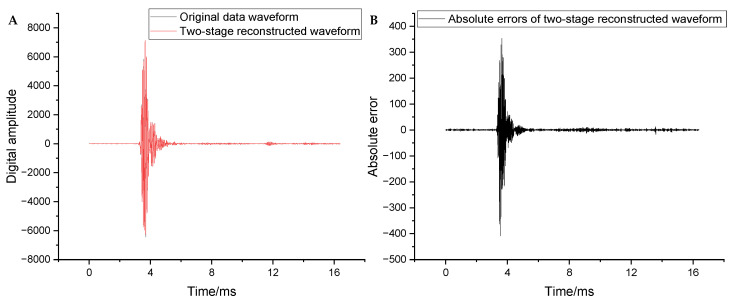
(**A**) Comparison between the reconstructed and original waveforms. (**B**) Absolute errors of the reconstructed waveforms.

(1) Compression rate
  The storage size of the data was 2048 words. After one layer of wavelet transform compression, the size was reduced to 1027 words, resulting in a first-stage compression rate of 50.15%. Following the second-stage ADPCM compression, the data were further reduced to 1028 bytes, achieving an overall compression rate of 25.1%. Notably, the compression rate of this two-stage compression algorithm is independent of the hardware platform and is determined only by the algorithm itself.

(2) Distortion
  The distortion of the full waveform was evaluated from the global and local perspectives. Figure 8A compares the original data with the two-stage reconstructed waveforms. The black and blue curves represent the original and two-stage reconstructed waveform, respectively. The absolute error curve of the two-stage reconstructed waveforms is shown in Figure 8B. The overall restoration effect of the reconstructed waveform is good and well retains the morphology of the original waveform. The distortion was large only in the sliding waves with larger amplitudes, whereas it remained small in all the other positions. The maximum absolute error (407.9) occurred at 3.552 ms, where the original data was 445 and the reconstructed data was 852.9, with a relative error of approximately 90%. The mean square error (MSE) was 32.17.

**Figure 9:**
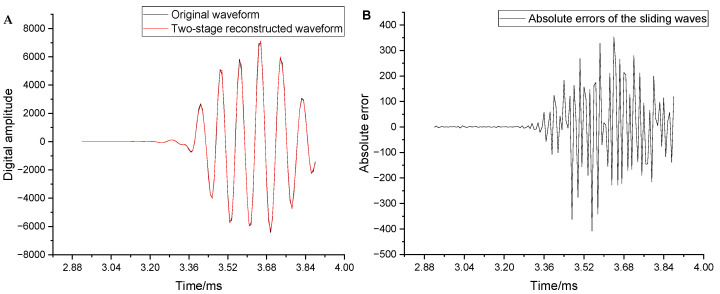
(**A**) Comparison of the waveforms near the arrival of the sliding waves. (**B**) Absolute errors near the arrival of the sliding waves.

**Figure 10:**
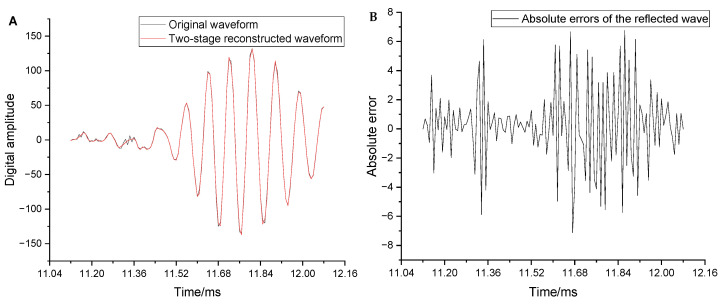
(**A**) Comparison between the original and reconstructed waveforms near the arrival of the reflected wave. (**B**) Absolute errors corresponding to the reconstructed waveform in the same region.

To further evaluate the distortion of local key positions in the full waveform, the wave packet data near the arrival of the sliding waves and the arrival of the reflected wave within the full waveform were analyzed. Each wave packet contained 60 data points. Figure 9 compares the original and reconstructed waveforms, along with their absolute errors near the arrival of the sliding waves. The overall reconstruction of the waveform near the arrival of the sliding waves was observed to be extremely good, thus enabling effective extraction of the sliding longitudinal and transverse waves. The maximum relative distortion of individual isolated points (Figure 8B) did not affect the overall waveform shape. Figure 10 compares the original and reconstructed waveforms along with the absolute errors near the arrival of the reflected wave. The overall reconstruction of the waveform in this region was also observed to be good. The maximum absolute error was only 7.1, and the maximum relative distortion did not exceed 5%. This indicates that the remote-detection reflected wave can be easily extracted.

**Figure 11:**
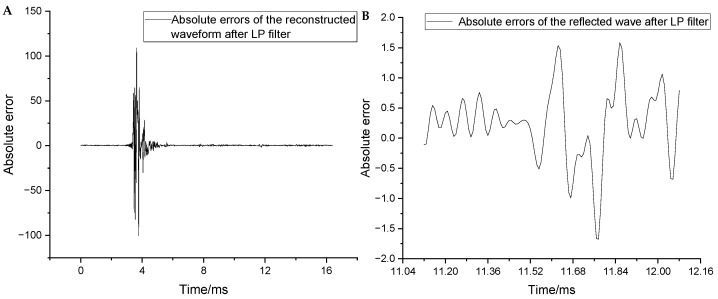
(**A**) Absolute errors of the reconstructed waveforms after low-pass filtering. (**B**) Absolute errors after low-pass filtering near the arrival of the reflected wave.

To analyze the distortion of useful frequency components (i.e., below 20 kHz), the original and reconstructed waveforms were passed through a low-pass filter with a cut-off frequency of 20 kHz. The distortion parameters were then re-evaluated. Figure 11A shows the absolute errors of the full waveform after low-pass filtering. The maximum absolute error, maximum relative error, and MSE decreased to 109.2, 1.5%, and 8.81, respectively. Figure 11B shows the absolute errors near the arrival of the reflected wave after low-pass filtering. The maximum absolute error in this region decreased from 7.1 to 1.66. These results confirm that the distortion in the two-stage reconstructed waveform was primarily due to the loss of high-frequency information. In contrast, the reconstruction of low-frequency information was significantly improved. In summary, the reconstruction distortion in the full waveform did not impede the extraction of valid information from the logging data.

**Figure 12:**
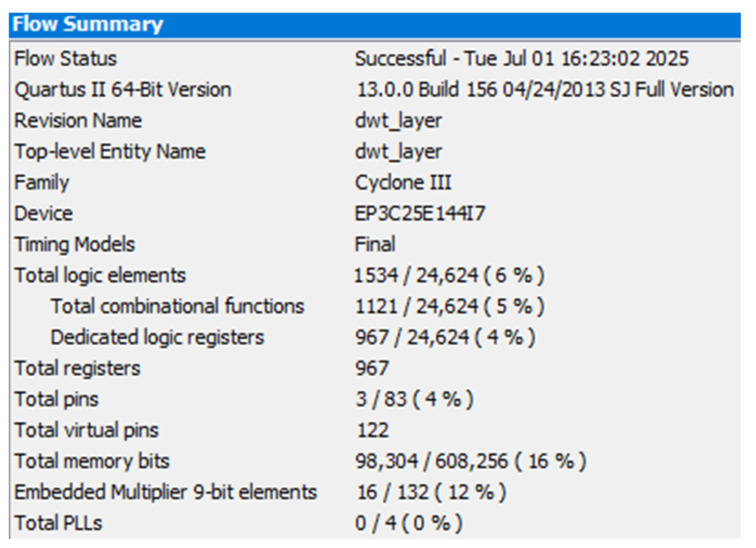
Resource utilization in the FPGA.

**Figure 13:**
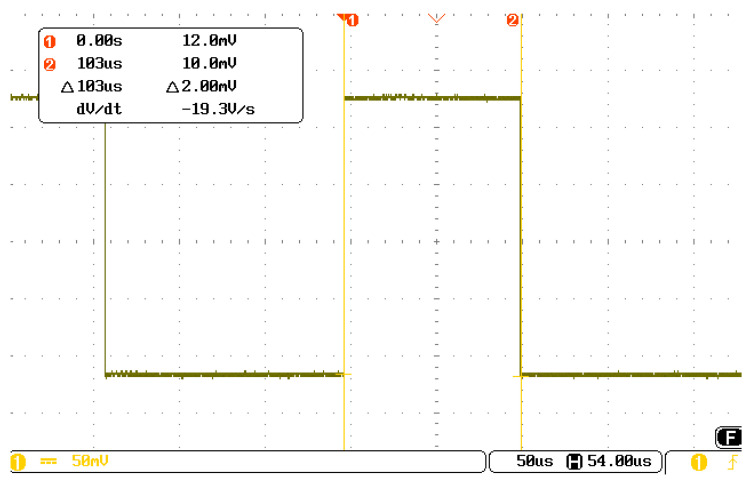
Execution time of the two-stage compression algorithms in the FPGA.

(3) Resource usage
  Figure 12 shows the FPGA resource utilization of the two-stage compression algorithm. The algorithm uses 16% of the memory resources and 12% of the multiplier resources in the FPGA, and all the other resources are relatively low. The multiplier resource is heavily used because of the numerous convolution operations in the wavelet-transform-based first-stage compression. The high memory usage stems from the algorithm compression of 2048 16-bit data in two stages, which involves several caching modules, such as the ROM, FIFO and RAM.

(4) Execution time
  The execution time of the two-stage compression algorithm under the above conditions was evaluated using an oscilloscope. The results are shown in Figure 13. The time taken to run the algorithm in the FPGA was approximately 103 µs, which is the same as the simulation results.

### 4.2. Applications Analysis in Remote-Detection Acoustic Logging

In the current remote-detection acoustic logging tools, the substantial data collected at each depth places a heavy workload on the DSP of the master control node, which simultaneously executes tool control, data uploading and many algorithms. This results in long execution times, which is unfavorable for quick logging. Previous research has demonstrated that the execution time of compression using a one-layer wavelet transform or ADPCM on a downhole DSP of TMS320F28335, which can work stably at 175 °C, is approximately 5 ms [6]. This indicates that only compression algorithms with short execution times and low resource utilization are feasible in the DSP for remote-detection acoustic logging tools. This severely limits the downhole data compression efficiency and cable transmission enhancement.

Simultaneously, each receiving node in the remote-detection acoustic logging tool uses an FPGA as the acquisition controller, providing a hardware foundation for implementing data compression at the acquisition node. Moreover, this study demonstrates that the execution time of the two-stage compression algorithm based on wavelet transform and ADPCM is approximately 100 μs in EP3C25E144I7, which is considerably faster than the 10 ms level in the DSP and can work stably at 175 °C. The execution time advantage is obvious. In addition, the algorithm exhibits low resource usage in an FPGA.

If successfully implemented in remote-detection acoustic logging tools, the two-stage data compression method at the acquisition node can reduce the data transmission to the master-controlled DSP and to the ground to 25% of the original amount, which is better than the achieved compression rate of 50% at the master control node [17]. This can extensively reduce the time required for logging data travelling in the tool and uploading to the ground and the workload of the DSP on the master control node, thereby increasing the logging speed to 400%.

## 5. Conclusions

To enhance the efficiency of cable transmission and reduce the workload at the master control node, we proposed a two-stage compression method at the acquisition node for remote-detection acoustic logging. We designed FPGA-based controller hardware and developed software for the two-stage compression at the acquisition node and ground decompression. We then tested the performance of the proposed algorithm using actual logging data. The following conclusions can be drawn:

(1) The compression rate of the two-stage compression method based on the one-layer wavelet transform and the ADPCM algorithm was 25.1% for remote-detection acoustic logging data. The reconstructed waveforms well preserved the morphology of the original waveforms. Even with remarkable relative distortion in individual data points, the extraction of longitudinal, transverse and reflected waves remained unaffected.
(2) The execution time of the two-stage compression for 2048 16-bit waveform data was approximately 100 µs in an FPGA with low resource usage and a light workload. The proposed compression performance considerably outperforms DSP-based compression methods.
(3) The proposed data compression method can reduce the volume of data transmitted from the acquisition node to the master control node and then uploaded to the ground to 25% of the original amount. This substantially reduces the workload on the master control node, enhances the cable transmission efficiency and increases the logging speed to approximately 400%. This study provides a reference for designing next-generation remote-detection acoustic logging tools.

Further improvements in the compression efficiency and real-time processing performance are required to enhance the overall effectiveness of the proposed method. First, the compression rate and execution time should be optimized. For example, segment-wise ADPCM processing may improve the compression rate, and increasing the running clock frequency using the FPGA’s PLL module can reduce the algorithm’s execution time for multi-channel signal processing. Second, the integration of the two-stage compression functionality into existing acquisition nodes will be essential for practical field deployment.

## Figures and Tables

**Table 1:** Data field definitions of the interval quantization and adaptive encoding.

Range of Absolute Difference	Sign Code Field	Segment Code Field	Segment Value	Interval Code Field	Interval Value	Max Absolute Error	Max Relative Error (%)
0–7	bit 7	bit 6–3	8	bit 2–0	0–7	0	0
8–15	bit 7	bit 6–3	9	bit 2–0	0–7	0	0
16–23	bit 7	bit 6–3	10	bit 2–0	0–7	0	0
24–31	bit 7	bit 6–3	11	bit 2–0	0–7	0	0
32–47	bit 7	bit 6–3	12	bit 2–0	0–7	1	3.125
48–63	bit 7	bit 6–3	13	bit 2–0	0–7	1	2.083
64–95	bit 7	bit 6–3	14	bit 2–0	0–7	2	3.125
96–127	bit 7	bit 6–3	15	bit 2–0	0–7	2	2.083
128–255	bit 7	bit 6–4	0	bit 3–0	0–15	4	3.125
256–511	bit 7	bit 6–4	1	bit 3–0	0–15	8	3.125
512–1023	bit 7	bit 6–4	2	bit 3–0	0–15	16	3.125
1024–2047	bit 7	bit 6–4	3	bit 3–0	0–15	32	3.125
2048–4095	bit 7	bit 6–4	4	bit 3–0	0–15	64	3.125
4096–8191	bit 7	bit 6–4	5	bit 3–0	0–15	128	3.125
8192–16,383	bit 7	bit 6–4	6	bit 3–0	0–15	256	3.125
16,384–32,767	bit 7	bit 6–4	7	bit 3–0	0–15	512	3.125

## Data Availability

The raw data supporting the conclusions of this paper will be made available by the authors upon request.
